# Dengue Maculopathy with Foveolitis in a Postpartum Female

**DOI:** 10.7759/cureus.1942

**Published:** 2017-12-13

**Authors:** Kok Wei Kan, Patrick Sylves, Nik-Lah Nik-Ahmad-Zuky, Ismail Shatriah

**Affiliations:** 1 Department of Ophthalmology, School of Medical Sciences, University Science Malaysia, Kubang Kerian, Kelantan, Malaysia; 2 Department of Obstetric and Gynecology, School of Medical Sciences, University Science Malaysia, Kubang Kerian, Kelantan, Malaysia

**Keywords:** postpartum, dengue fever, maculopathy, foveolitis, methylprednisolone, immunoglobulin

## Abstract

Dengue fever is common in the tropics and its clinical manifestations and complications are well-known. However, dengue-related ocular complications are rare. Here we present a postpartum female who complained of bilateral central scotoma, at five days after the clinical diagnosis of dengue fever. The ocular examination was suggestive of dengue maculopathy and foveolitis. She was treated with a combination of intravenous methylprednisolone and immunoglobulin. The final visual recovery was good.

## Introduction

Dengue fever is known to affect a few of the organ systems, but ocular involvement is rare. The ocular involvement was only seen in 10% of the cases, with a wide variety in the presentation, from subtle signs like subconjunctival hemorrhage, uveitis, vitreous hemorrhage, retinal hemorrhages, to blinding complications such as maculopathy, foveolitis, macular edema, retinal vascular occlusion, and optic neuropathy [1-2]. These ophthalmic complications are usually seen in young adults who are often present at the nadir of thrombocytopenia [3].

The occurrence of dengue fever was demonstrated in 2.5% of the participants in a study conducted by Tan, et al. [4]. We reported a postpartum female who had dengue maculopathy and foveolitis and was successfully treated using a combination of intravenous methylprednisolone and immunoglobulin.

## Case presentation

A 33-year-old female was admitted for dengue fever at day 30 postpartum based on dengue immunoglobulin M (IgM) detection in her blood. Her pre-morbid vision was satisfactory with the help of low myopic glasses. Her ocular symptoms started on day five of dengue fever, including sudden onset painless loss of the vision in both the eyes upon awakening in the morning. The loss of vision was more pronounced in the left eye and was associated with central scotoma. There was no metamorphopsia, floaters or photopsia, and no pain on eye movement, eye discharge or redness of the eye. 

On admission, her body temperature was 37 degrees Celsius, blood pressure was 110/80 mmHg, pulse rate was 70/min, and respiratory rate was 18/min. Hydration was adequate with normal breathing and heart sound. There was no evidence of bleeding tendency. The systemic examination was normal.

**Figure 1:**
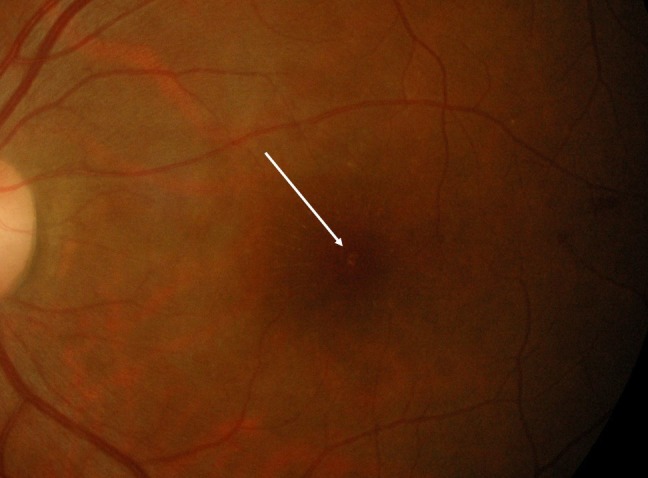
The color fundus photograph of the left eye showing a yellowish-orange lesion at the fovea.

Best corrected visual acuity (BCVA) was 6/24 in the right eye and 1/60 in the left eye. The relative afferent pupillary defect was present in the left eye. The optic nerve examination tests revealed a positive red desaturation and reduced light sensitivity in the left eye. There was an absence of petechial hemorrhages, conjunctival hemorrhages, and subconjunctival hemorrhages. The anterior chamber was deep and quiet. The funduscopic examination revealed a yellowish-orange lesion at the fovea on both eyes. There was a resolving macular star in the left eye (Figure 1). No retina or vitreous hemorrhage was noted. 

**Figure 2:**
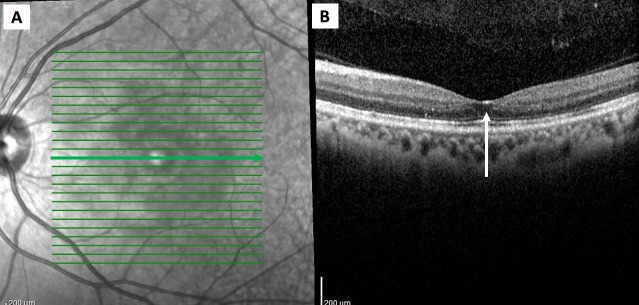
The optical coherence tomography (Heidelberg) of the left eye demonstrating the subfoveal nodule with the elevation of the inner retinal surface.

The refraction revealed low myopia of -2.00 diopters in both eyes. The visual field assessment demonstrated central scotoma in the left eye. The optical coherence tomography (OCT) showed the presence of sub-foveal nodule and multiple exudates, with signs of disruption of the outer retina (Figure 2). The magnetic resonance imaging of the brain and orbit showed no signs suggestive of the optic neuritis.

She was diagnosed with dengue maculopathy and foveolitis in both eyes. Full blood count, renal function, and the liver function tests were normal. She was started on intravenous methylprednisolone 1 gram/day for three days, followed by oral prednisolone for 11 days. The BCVA in the right eye improved to 6/9 while the left eye remained at 1/60 at five days after initiation of the intravenous methylprednisolone.

The BCVA in the left eye showed no improvement on day seven of the treatment. Thus, the patient was started on intravenous immunoglobulin 400 mg/kg for five days. Her visual acuity started to improve on day three of immunoglobulin therapy. Upon completion of the immunoglobulin regime, her visual acuity in the left eye improved to 6/18 with the reduction in the size of central scotoma and improved red desaturation and light sensitivity. The BCVA in the right eye improved to 6/6.

**Figure 3:**
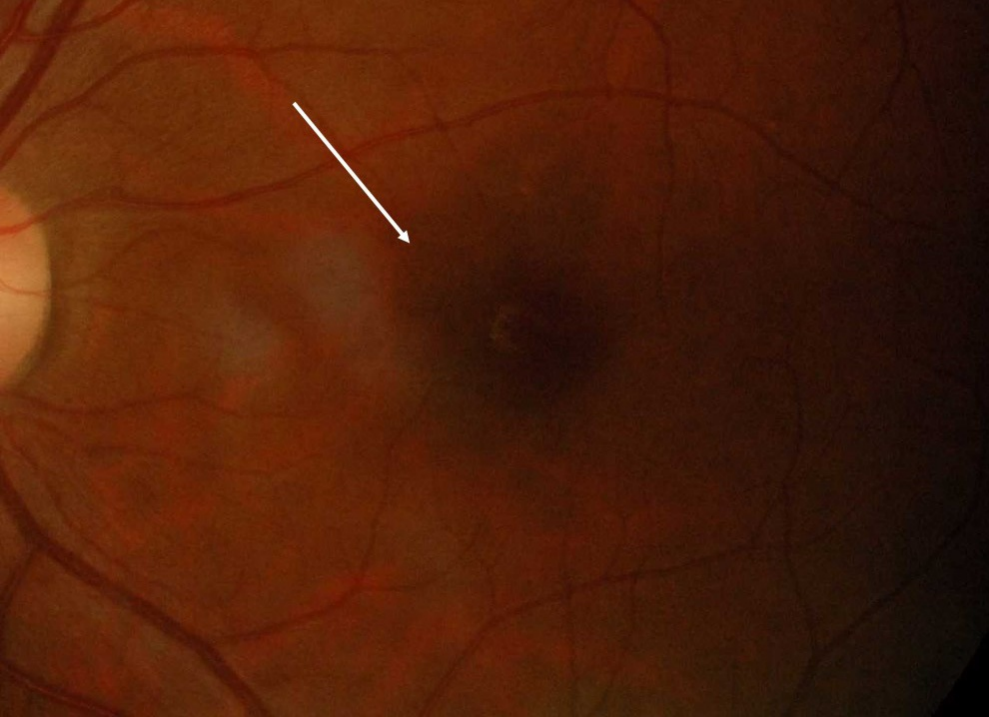
The color fundus photograph of the left eye four months after the treatment showing resolution of the subfoveal nodule.

**Figure 4:**
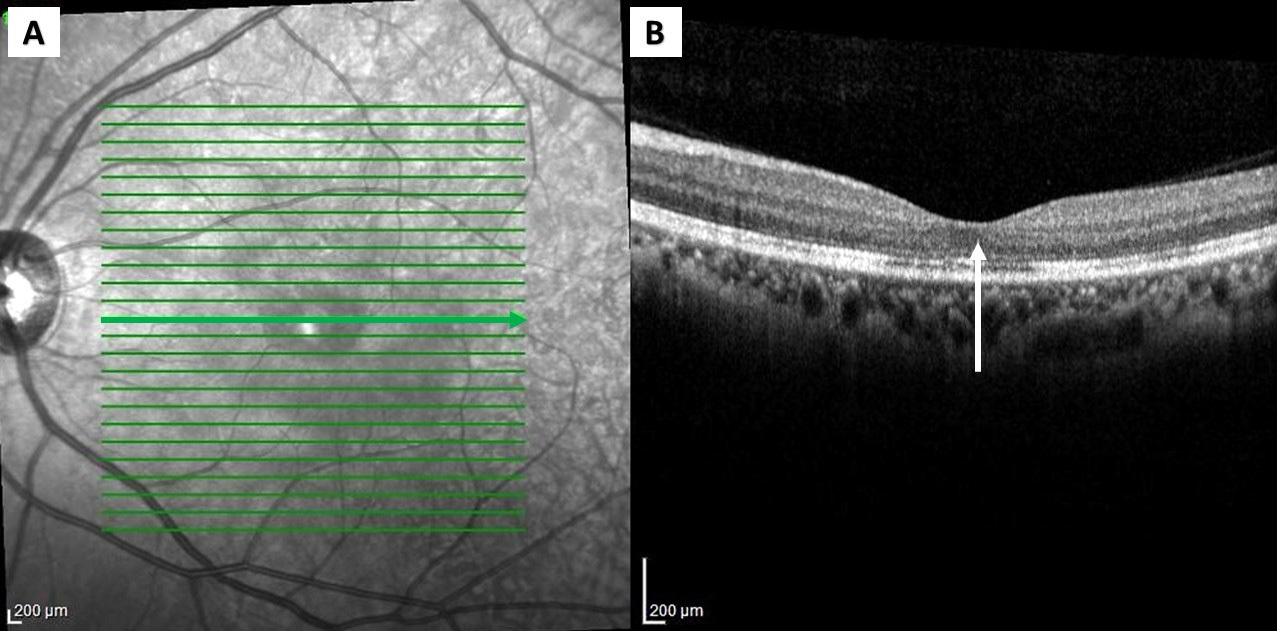
The optical coherence tomography (Heidelberg) of the left eye four months after the treatment showing the resolution of the subfoveal nodule and the improvement of the inner retinal surface.

Four months after the attack, the BCVA in the right eye was 6/6, while the left eye remained at 6/18 with the residual central scotoma. There was the resolution of the relative afferent pupillary defect in the left eye. Both red desaturation and light sensitivity tests had improved to 90% when compared to the right eye. Fundus photography revealed a resolved subfoveal nodule and macular star (Figure 3). The repeat OCT demonstrated improvement of the disruption of the outer retina at the fovea area (Figure 4).

## Discussion

The blurring of the vision is the most common complaint in the individuals affected by dengue-related ocular complication, and it is usually associated with maculopathy [3]. The visual acuity tends to be worse when there is dengue maculopathy associated with foveolitis. Our patient was diagnosed with dengue maculopathy with foveolitis, as evident upon fundus examination and OCT imaging. However, fundus angiography was not performed in our patient because she was breastfeeding her newborn during the attack.

Foveolitis, which corresponds to disruption of the outer neurosensory retina in the OCT can present a BCVA range from 6/6 to counting fingers in different reports [1,3,5-6]. They are usually present on day seven after the onset of the dengue fever [2]. This five to seven-day delay favors the hypothesis by Lim, et al., in which dengue-related ocular complications could be an immune-mediated process rather than directly due to the viral infection [7]. This is because, this period of defervescence corresponds to the time-of-onset of the antibody production, immune-complex deposition, or production of autoantibodies [5]. However, the exact immunological mechanism is still not completely understood.

In view of the immune-mediated hypothesis, the corticosteroid is the mainstay of the treatment in the patients with dengue foveolitis presented with poorer presenting BCVA unless there are contraindications. Intravenous methylprednisolone was started in our patient after a lengthy discussion with our patient regarding her timing for lactation. The treatment had to be modified in order to minimize the passing of medication through breast milk from the mother to her infant.

Bacsal, et al. described three patients who had persistent poor BCVA following intravenous corticosteroid treatment [8]. Subsequently, the patients were treated with immunoglobulin and their visual acuity had improved [8]. Our patient demonstrated a similar clinical phenomenon with minimal improvement of the visual acuity after the initial corticosteroid. She showed significant visual improvement in both eyes following a combination of intravenous methylprednisolone and immunoglobulin therapy.

The amount of methylprednisolone presence in the breast milk of a lactating mother treated with high intravenous doses of methylprednisolone, such as one gram used in our patient is very low. Fully breastfed infants would receive doses nearing their daily cortisol output, but less than a therapeutic dose, on the day of infusion. Therefore, it is important to avoid breastfeeding during the infusion for two to eight hours after a one gram dose [6, 9]. The immunoglobulin is a normal component of breastmilk and has been reported safe to be used for lactating mothers [10].

Bacsal, et al. reported that the patients with dengue maculopathy with foveolitis had poorest visual prognosis [8]. In the study, all the patients diagnosed with foveolitis noticed a scotoma even after the clinical and anatomical structural resolution. This is thought of, as a result of the inflammatory disturbance of the outer retina in the subfoveal region [8]. Similarly, our patient still complained of the persistent residual scotoma at four months after the attack.

## Conclusions

Dengue fever with maculopathy and foveolitis is an uncommon manifestation, especially during the postpartum period. Immune modulators are used to treat the patients with poor presenting visual acuity. By advising a change in the breastfeeding habit, a combination of intravenous methylprednisolone and immunoglobulin can be safely administrated to a lactating mother with the improved visual outcome.
